# Light-Addressed Electrodeposition of Enzyme-Entrapped Chitosan Membranes for Multiplexed Enzyme-Based Bioassays Using a Digital Micromirror Device

**DOI:** 10.3390/s130810711

**Published:** 2013-08-16

**Authors:** Shih-Hao Huang, Lu-Shiuan Wei, Hsiao-Tzu Chu, Yeu-Long Jiang

**Affiliations:** 1 Department of Mechanical and Mechatronic Engineering, National Taiwan Ocean University, Keelung 202-24, Taiwan; E-Mails: killer30614@msn.com (L.-S.W.); a90411_5@hotmail.com (H.-T.C.); 2 Center for Marine Mechatronic Systems, CMMS, National Taiwan Ocean University, Keelung 202-24, Taiwan; 3 Graduate Institute of Optoelectronic Engineering, Department of Electrical Engineering, National Chung Hsing University, Taichung 402-27, Taiwan; E-Mail: yljiang@nchu.edu.tw

**Keywords:** electrodeposition, digital micromirror device, chitosan, enzyme-based bioassay

## Abstract

This paper describes a light-addressed electrolytic system used to perform an electrodeposition of enzyme-entrapped chitosan membranes for multiplexed enzyme-based bioassays using a digital micromirror device (DMD). In this system, a patterned light illumination is projected onto a photoconductive substrate serving as a photo-cathode to electrolytically produce hydroxide ions, which leads to an increased pH gradient. The high pH generated at the cathode can cause a local gelation of chitosan through sol-gel transition. By controlling the illumination pattern on the DMD, a light-addressed electrodeposition of chitosan membranes with different shapes and sizes, as well as multiplexed micropatterning, was performed. The effect of the illumination time of the light pattern on the dimensional resolution of chitosan membrane formation was examined experimentally. Moreover, multiplexed enzyme-based bioassay of enzyme-entrapped chitosan membranes was also successfully demonstrated through the electrodeposition of the chitosan membranes with various shapes/sizes and entrapping different enzymes. As a model experiment, glucose and ethanol were simultaneously detected in a single detection chamber without cross-talk using shape-coded chitosan membranes entrapped with glucose oxidase (GOX), peroxidase (POD), and Amplex Red (AmR) or alcohol oxidase (AOX), POD, and AmR by using same fluorescence indicator (AmR).

## 1. Introduction

Chitosan is a naturally derived polymer obtained from the deacetylation of chitin, which is an abundant, biocompatible and biodegradable biomaterial. Due to its pH dependent solubility, porous structure, and dense amine groups, chitosan films can be fabricated and integrated into devices by several methods compatible with standard microfabrication technology, including solution casting, spin casting, electrodeposition, and nanoimprinting. The formed chitosan films are well-suited as a matrix for the attachment of biomolecules, such as proteins, enzymes, antibodies, and DNA [1–3]. Among them, electrodeposition is an attractive technique for depositing pH-responsive chitosan at specific addresses and in specific shapes on the preformed metal thin-film electrode surfaces that can generate hydroxide ions (OH^−^) by electrolysis on the cathodes of the electrodes [4–7]. This technique allows for both spatial and temporal control of the chitosan film location and thickness, and it can be used with a variety of device geometries. However, gel patterns involving locations, shapes and dimensions are completely subject to the pre-defined configurations of microelectrodes. Changing the pattern requires the re-design and re-fabrication of the photo-masks, microelectrodes and sometimes the chip structures themselves. This inflexibility of the microelectrodes has hampered the development of biological applications requiring dynamic and multiplexed micropatterning of chitosan membranes for the attachment of various biomolecules on the same device.

The electrical co-deposition of chitosan with biomolecules, such as proteins, enzymes, antibodies, and DNA has been successfully demonstrated for biosensors [8–13]. Chitosan films can be also used for the immobilization of microbial cells and further sensing of their respiratory activity [14]. Powers *et al.* developed a multipurpose optical detection platform with an electrodeposited chitosan film for detection of DNA hybridization [9]. Zhu *et al.* reported micromachined amperometric sensors for glucose, glutamate, and galactose made using chitosan membranes [15]. The devices have a pyramidal chamber array anisotropically etched in silicon. Each chamber contains a platinum electrode at the bottom covered with a solution-cast chitosan membrane. The membranes are modified with the enzymes glucose oxidase or galactose oxidase by means of glutaraldehyde crosslinking. Although these devices using chitosan films for biomolecule attachment have the capability of detecting one analyte in a single microchannel/microchamber, the simultaneous detection of different analytes could not be easily achieved. Multiplexed assays require multiple microchannels/microchambers, or multiple fluorescence indicators, or complicated channel designs.

In our previous work [16], we proposed for the first time a light-addressable electrolytic system used to perform an electrodeposition of calcium alginate hydrogels using a digital micromirror device (DMD). In this system, a patterned light illumination is projected onto a photoconductive substrate serving as a photo-anode to electrolytically produce protons, which can lead to a decreased pH gradient. The low pH generated at the anode can locally release calcium ions from insoluble calcium carbonate (CaCO_3_) to cause gelation of calcium alginate through sol-gel transition. Here, we propose a light-addressed electrolytic system used to perform an electrodeposition of enzyme-entrapped chitosan membranes for multiplexed enzyme-based bioassay using a DMD. The DMD provides spatio-temporal illumination pattern switching, which is used to achieve flexible electrode patterning for the dynamical and multiplexed electrodeposition of chitosan membranes. Changing the desired patterns does not require the re-design and re-fabrication of the photo-masks. By controlling the illumination pattern on the DMD, a light-addressed electrodeposition of chitosan membranes with different shapes and sizes, as well as multiplexed micropatterning, was performed. For conventional microfluidic or microparticle-based enzyme assays, a multiplexed assay requires multiple microchannels, or multiple fluorescence indicators, or complicated channel designs. In this study, multiplexed enzyme-based bioassay of enzyme-entrapped chitosan membranes was demonstrated through the electrodeposition of the chitosan membranes with different shapes and entrapping different enzymes for the purpose of simultaneously detecting multiple analytes by using same fluorescence indicator within a single microchamber. As a model experiment, glucose and ethanol were simultaneously detected in a single detection chamber using shape-coded chitosan membranes with entrapped glucose oxidase (GOX), peroxidase (POD), and Amplex Red (AmR) or alcohol oxidase (AOX), POD, and AmR by using same fluorescence indicator (AmR).

## 2. Experimental Section

### 2.1. Light-Addressed Electrodeposition of Enzyme-Entrapping Chitosan Membranes

**Figure 1.**
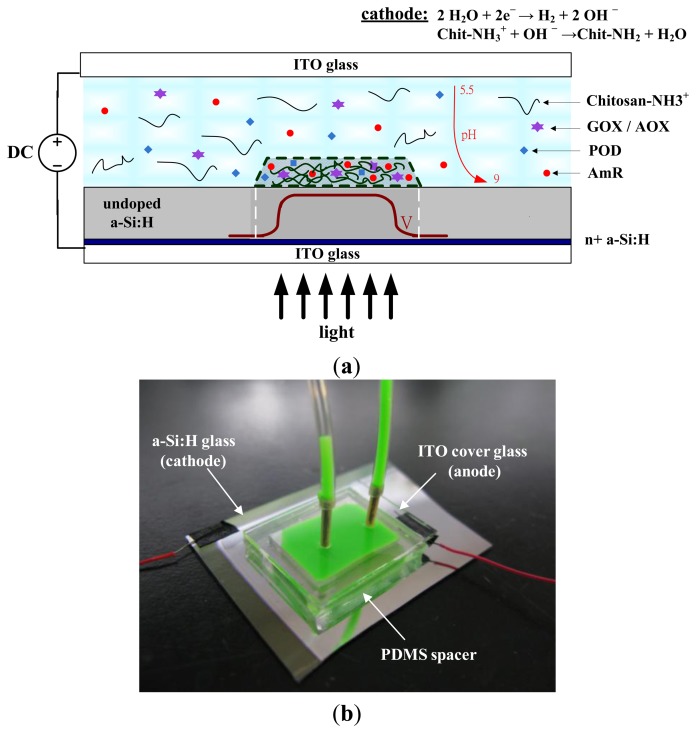
(**a**) Schematic diagram of a light-addressed electrolytic system for performing the electrodeposition of enzyme-entrapped chitosan membranes using a DMD. (**b**) An image of the PDMS spacer bonded to the photoconductive substrate, and an ITO cover glass serving as the anode used to seal the microchamber (green dye was introduced into the microchamber for visibility purposes).

Figure 1 shows a light-addressed electrolytic system used to perform an electrodeposition of enzyme-entrapping chitosan membranes using a DMD. A photoconductive substrate, which consists of 0.2 μm of heavily doped hydrogenated amorphous silicon (n^+^ a-Si:H) and 1 μm of undoped a-Si:H on a 700-μm indium tin oxide (ITO) glass substrate, serves as a light-addressable photo-cathode. A polydimethylsiloxane (PDMS) spacer with 0.5 mm in height was bonded to the photoconductive substrate, and an ITO cover glass that serves as the anode was used to seal the spacer to form a microchamber. The deposition solution, which contains soluble chitosan either mixed with GOX, POD, and AmR or AOX, POD, and AmR, was introduced into the microchamber (Figure 1a). A cathode voltage was applied to the photoconductive substrate via the ITO layer, and ITO cover glass was served as the anode. Figure 1b shows the image of the PDMS spacer bonded to the photoconductive substrate, and an ITO cover glass/anode used to seal the microchamber (green dye was introduced into the microchamber for visibility purposes).

When a DC voltage is applied concurrently, light illumination on a photoconductive substrate generates a conducting point that acts as a photo-cathode to electrolytically produce hydroxide ions (OH^−^), which can lead to an increased pH gradient (Equation (1)). The high pH generated at the cathode can locally cause gelation of chitosan through sol-gel transition (Equation (2)):

(1) $$2\;{\text{H}}_{2}\text{O}+2\;{\text{e}}^{-}\to {\text{H}}_{2}+2\;{\text{OH}}^{-}$$

(2) $$\text{Chit}-{{\text{NH}}_{3}}^{+}+{\text{OH}}^{-}\to \text{Chit}-{\text{NH}}_{2}+{\text{H}}_{2}\text{O}$$

Chitosan is an amino-polysaccharide (Chit–NH_3_^+^) that under moderately acidic conditions (pH < 6) is a soluble, cationic polyelectrolyte. When the pH is raised above about 6.3, the amino groups become deprotonated and this polysaccharide can form an insoluble hydrogel network (Chit-NH_2_). During this process, the hydrogel network provides a suitable matrix for the attachment of enzymes (GOX, AOX, POD, and AmR) within the deposition solution to form a stable film of enzyme-entrapped chitosan membranes. GOX and AOX were chosen as the model oxidases and Amplex Red was used as the fluorescence indicator in this study. Amplex Red is a very stable and sensitive fluorogenic substrate for POD. In the presence of POD, Amplex Red reacts with hydrogen peroxide with a 1:1 stoichiometry to produce highly fluorescent resorufin [17,18]. Multiplexed enzyme-based bioassay of enzyme-entrapped chitosan membranes was demonstrated through the electrodeposition of the chitosan membranes with various shapes and entrapping different enzymes. As a model experiment, glucose and ethanol were simultaneously detected using shape-coded chitosan membranes entrapped with GOX/POD/AmR (circle) and AOX/POD/AmR (square) by using same fluorescence indicator (AmR) in a single detection chamber.

### 2.2. Experimental Setup for a Light-Addressed Electrolytic System

**Figure 2.**
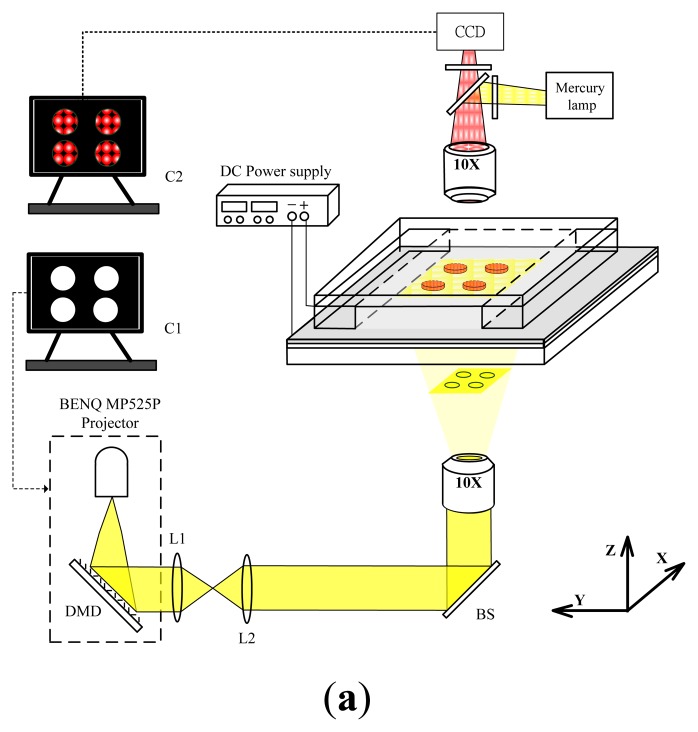

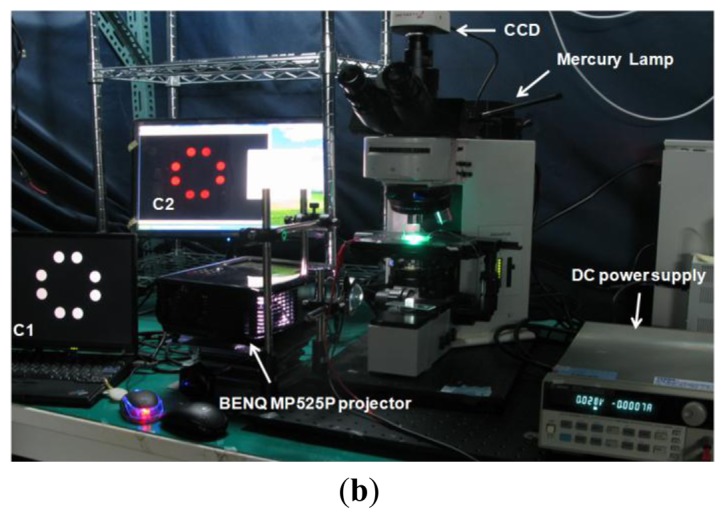
(**a**) Schematic diagram and (**b**) photograph of an experimental setup for a light-addressed electrolytic system for performing the electrodeposition of enzyme-entrapped chitosan membranes. A modified commercial DMD projector that is equipped with a mercury lamp unit as the light source. (L1 and L2: focus lens, BS: beam splitter).

Figure 2 shows an experimental setup for a light-addressed electrolytic system for performing electrodeposition of enzyme-entrapped chitosan membranes. To observe the illumination pattern, and pH-dependent fluorescent images, a standard upright BX51fluorescence microscope (Olympus, Tokyo, Japan) was used with a charge coupled device (CCD) camera (INFINITY2-1, Lumenera, Ontario, Canada). Excitation light for fluorescence was projected onto the photoconductive substrate by a 10× objective lens through a filter unit, which consists of excitation and emission band-pass filters of 470–495 and 510–550 nm, respectively, and a dichroic mirror with cutoff at 505 nm. The pH-dependent fluorescent images were observed via a computer (C2). A DMD-based light-addressing projection optical system for was set up under the microscope stage. This setup uses a modified commercial MP525P DMD projector (BENQ, Hsinchu, Taiwan) that is equipped with a mercury lamp unit as the light source. We modified the commercial DMD projector by simply removing the projection lens and the color filter wheel [16]. The structured light patterns of the DMD were controlled by a computer (C1). The continuous light illuminated the DMD uniformly through the built-in condensing lens within the DMD projector and then spatially projected it onto the photoconductive substrate through a focus lens (L1 and L2), a 50/50 beam splitter (BS), and an objective lens with 10× magnification. A cathode voltage was applied to the photoconductive substrate via a 50 W 6613C DC power source (HP/Agilent, Santa Clara, CA, USA), and the ITO cover glass served as the anode. The voltage application was synchronized with the projection of an illumination pattern by the DMD. Focal light illumination locally increases the conductivity of the photoconductive substrate and thus creates a virtual photo-electrode with flexible addressability and patternability, which is a substitute candidate for conventional metal microelectrodes. We can control the illumination pattern on the DMD, which enables the performance of an electrodeposition of chitosan membranes with different shapes and sizes and multiplexed micropatterning.

### 2.3. Materials

Chitosan from crab shells (85% deacetylation) was purchased from Sigma-Aldrich Chemicals (St. Louis, MO, USA). Chitosan solutions were prepared by adding chitosan flakes to water and incrementally adding small amounts of HCl to the solution to maintain the pH near 3. After being mixed overnight, the chitosan solutions were filtered to remove undissolved material, and the pH of solution was adjusted using NaOH (1M). The pH of the collected fluid was measured using a PH-206 pH meter (Lutron, Taipei, Taiwan). Glucose oxidase (GOX, 50,000 unit/g), peroxidase (POD, 200 unit/mg), alcohol oxidase (AOX, 10–40 units/mg), β-d-glucose, and ethanol were also purchased from Sigma-Aldrich Chemicals. Amplex Red reagent and reaction buffer were purchased from Molecular Probes (Grand Island, NY, UAS). 2′,7′-bis-(2-carboxyethyl)-5-(and-6)-carboxyfluorescein (BCECF) was used as a fluorescence pH indicator [19]. The indicator was purchased from Sigma-Aldrich Chemicals and diluted to the final BCECF concentration of 0.2 μM before experiments. The pH-dependent fluorescence intensity of BCECF was calibrated by the pure DI water, which was adjusted by HCl/NaOH to maintain the pH at 5, 5.5, 6.5, 7, 8 and 9 with the above-mentioned imaging setup.

## 3. Results and Discussion

### 3.1. pH-Dependent Fluorescent Images of the Light-Addressed Cathode Electrolysis

**Figure 3.**
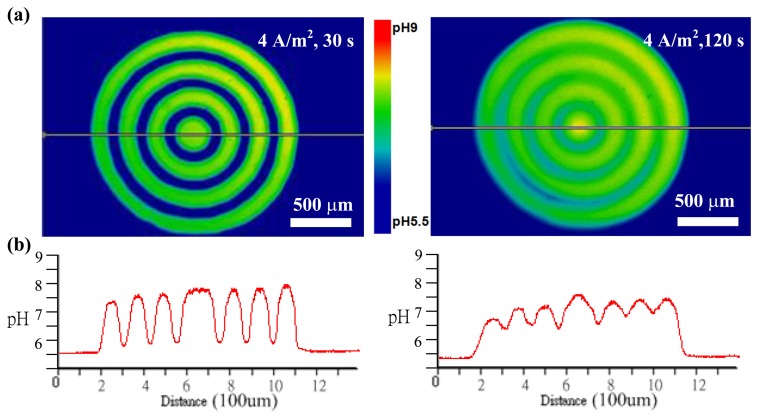
(**a**) The pH-dependent fluorescent images and (**b**) the cross-section profiles of the pH distribution in (a) when a patterned light illumination from DMD was projected onto a photoconductive substrate at an application of a current density of 4 A·m^−2^ for 30 s and 120 s, respectively.

To examine the feasibility of the light-addressed electrodeposition of chitosan membranes with different shapes and sizes, we first measured the pH-dependent fluorescent images at photo-cathodes when a patterned light illumination from the DMD was projected onto a photoconductive substrate. Pure DI water, which was adjusted by HCl to maintain the pH near 5.5, was introduced into the microchamber. A current density of 4 A·m^−2^ was applied to the device and synchronized with the projection of an illumination pattern with concentric circles by the DMD. Figure 3 shows the results of the pH-dependent fluorescent images and the cross-section profiles of pH distribution when applying the 4 A·m^−2^ current density for 30 s and 120 s, respectively. As shown in Figure 3a, pH-dependent fluorescent images with concentric circles were clearly observed around the photo-cathode surfaces, where hydroxide ions were generated. The pH-dependent fluorescent image at an applied current density of 4 A·m^−2^ for 30 s showed a high shape fidelity to the illumination patterns. The fluorescent image slightly became faint as the OH^−^ ions gradually diffuse when the illumination time increased from 30 s to 120 s. These results indicate that an increase in the illumination time for electrolysis results in an increase in OH^−^ ions produced from the photo-cathode. Figure 3b shows cross-section profiles of the pH distribution in Figure 3a. Seven peaks, where the pH values are larger than 6.3, correspond to the rings of the concentric circles. The results confirm that a patterned light illumination is projected onto a photoconductive substrate serving as a photo-cathode to electrolytically produce OH^−^ ions, which can lead to increased pH values larger than 6.3. The high pH (pH > 6.3) generated at the cathode can locally cause gelation of chitosan through sol-gel transition when a chitosan solution is introduced into the microchamber in these cases.

### 3.2. Light-Addressed Electrodeposition of Chitosan Membranes

**Figure 4.**
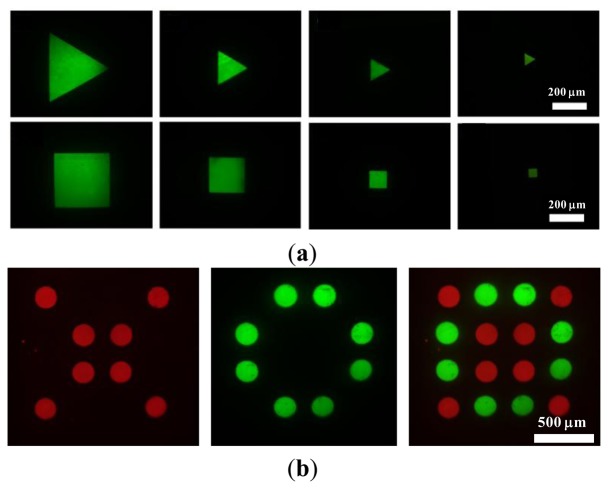
(**a**) Images of chitosan membranes with triangular and square shapes with different dimensions ranging from 50 μm to 300 μm. (**b**) The images of multiplexed micropatterning of chitosan membranes created by performing a light-addressed electrodeposition using two different illumination light patterns in sequence. The rightmost image shows the fluorescence micrograph of an assembled 4 × 4 microarray of chitosan membranes with a specific arrangement.

The flexible patternability of photo-electrodes would allow the generation of desired chitosan membrane patterns at a specific location, providing a great advantage over the electroaddressing of chitosan membranes using conventional metal microelectrodes. To demonstrate the feasibility of the light-addressed electrolytic system for performing the electrodeposition of chitosan membranes, chitosan membranes of different shapes and sizes and with multiplexed micropatterning were generated, as shown in Figure 4. In these experiments, the deposition solution, which contains soluble chitosan of 1% w/v and pH = 5.5, was introduced into the microchamber. FITC fluorescent dye (Sigma-Aldrich) was also blended into the deposition solution so that it was easy to observe. A current density of 4 A·m^−2^ was applied between the photoconductive substrate and ITO cover glass for 2.5 min. The illumination patterns on the DMD can be either triangular or square shapes with different dimensions ranging from 50 μm to 300 μm. The shaped light pattern was then projected onto the photoconductive substrate to electrolytically produce hydroxide ions (OH^−^) for the electrodeposition of chitosan membranes. The chitosan membranes deposited on the photoconductive layer were imaged using a fluorescence microscope coupled with a CCD to examine the shape fidelity between the chitosan membranes produced by the process and the illuminated DMD. As shown in Figure 4a, chitosan membranes were produced with 12 ± 3.2 μm in thickness, which was measured by a stylus-based surface profiler (KLA-Tencor/AS500, Milpitas, CA, USA) from three different samples, showed a high shape fidelity to the illumination patterns in either triangular or square shapes. We can successfully produce 50-μm-sized thin chitosan membranes on the photoconductive substrate after flushing with DI water. However, the chitosan membranes smaller than 50 μm were flushed away from the photoconductive substrate due to poor adhesion between the chitosan membranes and the photoconductive substrate. We also demonstrated the capability for multiplexed micropatterning to form a 4 × 4 microarray of chitosan membranes in circular shapes with a specific arrangement by performing light-addressed electrodepositions sequentially, as shown in Figure 4b. Two different deposition solutions mixed with two different fluorescent colors were sequentially electrodeposited using two different illumination light patterns. First we introduced the deposition solution of soluble chitosan (1% w/v, pH = 5.5) mixed with Rhodamine B (Sigma-Aldrich) into the microchamber.

The light-addressed electrodeposition was performed using an illumination pattern to produce the chitosan membranes with a red fluorescence, and then we repeated the electrodeposition to form chitosan membranes with a green fluorescent indicator (FITC) adjacent to the former using another illumination pattern. The rightmost image in Figure 4b shows the fluorescence micrograph of an assembled 4 × 4 microarray of chitosan membranes with a specific arrangement.

### 3.3. Characteristics of Light-Addressed Electrodeposition of Chitosan Membranes

**Figure 5.**
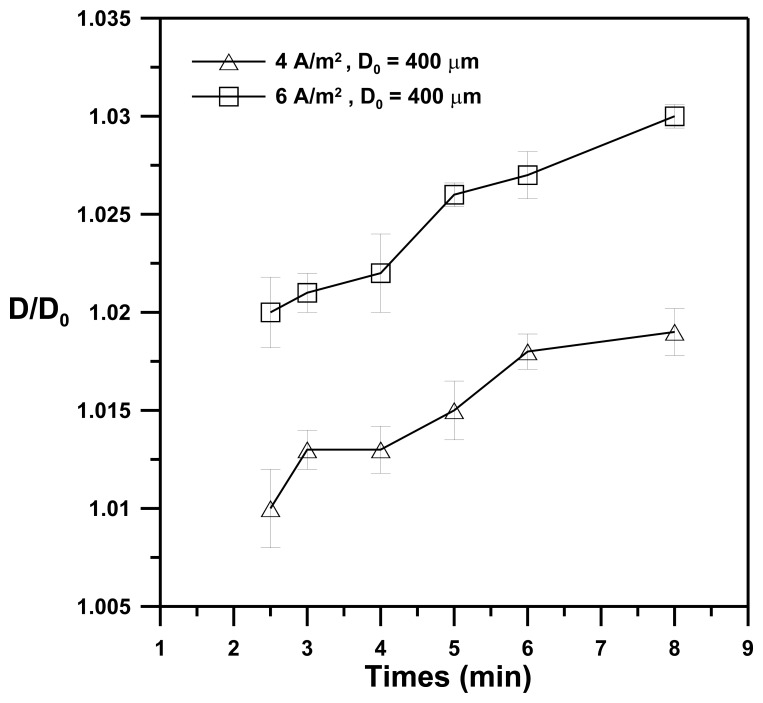
The effect of the illumination time of the light pattern on the dimensional resolution (D/D_0_) for a deposition solution (1% w/v of chitosan; pH = 5.5) with applied current densities of 4 and 6 A·m^−2^. (D_0_ and D denote the illumination pattern of circular shapes with D_0_ = 400 μm in diameter and the corresponding diameter (D) of the chitosan membranes produced by the illumination pattern).

Figure 5 shows the effect of the illumination time of the light pattern on the D/D_0_ ratio for the deposition solution (1% w/v of chitosan; pH = 5.5) with applied current densities of 4 A·m^−2^ and 6 A·m^−2^. The ratio of D/D_0_ is defined as the dimensional resolution, where Do and D denote an illumination pattern of circular shapes with D_0_ = 400 μm in diameter and the corresponding diameter (D) of the chitosan membrane produced by the illumination pattern, respectively. The data indicate the average value of five experiments, and the error bar shows the standard deviation under the same operating conditions. The D/Do ratio increases with increasing illumination time for applied current densities of both 4 A·m^−2^ and 6 A·m^−2^, whereas for a fixed illumination time the D/Do for an applied current density of 6 A·m^−2^ is larger than that for an applied current density of 4 A·m^−2^. These results indicate that an increase in the illumination time or in the applied current density for electrolysis results in an increase in OH^−^ ions produced at the photo-cathode. The OH^−^ ions diffuse and then react with the chitosan solution to produce chitosan membranes of larger sizes (D). These results demonstrate that chitosan membranes of varying sizes can be controllably electrodeposited by changing either the illumination time or the applied current densities.

### 3.4. Simultaneous Detection of Glucose and Ethanol

Multiplexed enzyme-based bioassay of enzyme-entrapped chitosan membranes was demonstrated through the electrodeposition of chitosan membranes with various shapes and entrapping different enzymes. As a model experiment, glucose and ethanol were simultaneously detected in a single detection chamber using shape-coded chitosan membranes entrapping GOX/POD/AmR (circle) and AOX/POD/AmR (square) by using same fluorescence indicator (AmR). Two different deposition solutions were sequentially electrodeposited using two different illumination light patterns. The deposition solution of chitosan (1% w/v) with GOX/POD/AmR was prepared by mixing Amplex Red stock solution (1%, v/v), GOX/POD stock solution (10%, v/v) with chitosan solution (89%, v/v). The final concentrations of Amplex Red, GOX, and POD in the mixture deposition solution were 50 μM, 300 units/mL, and 20 units/mL, respectively. Another deposition solution of chitosan (1% w/v) with AOX/POD/AmR was similarly prepared, except GOX was replaced by AOX. The chitosan membranes containing entrapped GOX/POD/AmR (circle) was first produced by introducing a deposition solution of chitosan mixed with GOX, POD, and AmR and performing an electrodeposition using an illumination pattern with four circles. Then we repeated the electrodeposition to form chitosan membranes entrapped with AOX/POD/AmR (square) adjacent to the former using an illumination pattern with five squares by introducing a deposition solution of chitosan mixed with AOX, POD, and AmR.

**Figure 6.**
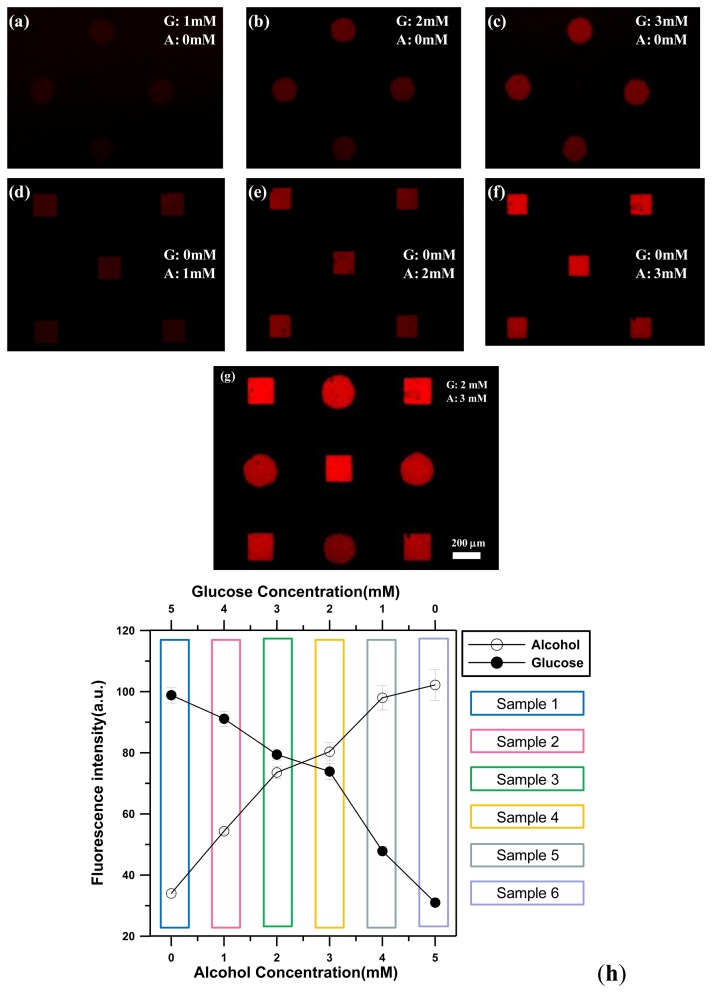
(**a**–**f**) Fluorescent images of respective detection of glucose or ethanol using shape-coded enzyme-entrapped chitosan membranes (circles: GOX/POD/AmR entrapped in chitosan membranes; squares: chitosan membranes with entrapped AOX/POD/AmR). Only the chitosan membranes entrapping a corresponding enzyme emitted red fluorescence without any cross-talk. (**g**) Fluorescent image and (**h**) intensity of simultaneous detection of glucose and ethanol using shape-coded enzyme-entrapped chitosan membranes, which were reacted with six different samples (each sample was composed of glucose and ethanol. G: glucose, A: alcohol).

To demonstrate the ability of glucose detection, the different glucose concentrations of 0, 1.0, 2.0, 3.0, 4.0, 5.0 mM were introduced into the microchamber, respectively. For chitosan membranes entrapped with GOX/POD/AmR (circles), when a glucose solution encountered the enzyme-entrapped chitosan membranes, glucose was converted into gluconolactone and hydrogen peroxide by the GOX. Non-fluorescent AmR then reacted with hydrogen peroxide in the presence of POD inside the chitosan membranes to produce fluorescent resorufin, as shown in Figure 6a–c. It is noted that no apparent fluorescence was observed for the square chitosan membranes with AOX, POD, and AmR under the glucose detection conditions. This result indicated that the presence of glucose solutions does not have any catalytic effect on the square chitosan membranes. For chitosan membranes with entrapped AOX/POD/AmR (squares), AOX can catalyze the chemical reaction with ethanol to produce the corresponding aldehyde and hydrogen peroxide. Similarly, the presence of hydrogen peroxide, AmR, and POD within the chitosan membranes led to the conversion of non-fluorescent AmR into fluorescent resorufin. To demonstrate the ethanol detection capability, different ethanol concentrations of 0, 1.0, 2.0, 3.0, 4.0, 5.0 mM were introduced into the microchamber. Only the square chitosan membranes with AOX, POD, and AmR emitted strong red light due to the bienzymatic reaction of AOX and POD, as shown in Figure 6d–f. Therefore, when a solution containing either glucose or ethanol was injected into a microchamber, only the chitosan membranes entrapping the corresponding enzyme emitted red fluorescence without any cross-talk. This indicated that the presence of one analyte does not have an effect on the other analyte for the case of glucose and ethanol.

To demonstrate the ability to simultaneously detect different concentrations of both analytes in a single microchamber, five different samples (mixtures of glucose and ethanol) were prepared by varying the molar ratio between the glucose and the ethanol (5:0, 4:1, 3:2, 2:4, 1:4, 0:5). The introduction of a solution containing both glucose and ethanol resulted in fluorescence from both shape-coded chitosan membranes entrapped with GOX/POD/AmR (circles) and AOX/POD/AmR (squares) (Figure 6g).

Figure 6h shows the changes in the fluorescence intensity from circular and square chitosan membranes when mixtures of glucose and ethanol with different molar ratios were injected into the microchamber. The data indicate the average value of three experiments, and the error bar shows the standard deviation under the same operating conditions. Both glucose and ethanol were successfully detected in the range of 0–5 mM and fluorescent intensity values from both shapes of chitosan membranes increased linearly with an increase of glucose or alcohol concentrations due to the production of more hydrogen peroxide.

## 4. Conclusions

In this study, we have proposed a novel electrolytic system for performing an electrodeposition of enzyme-entrapping chitosan membranes at desired locations on a photoconductive electrode substrate. Light illumination spatially patterned by a DMD produces a virtual photo-cathode (*i.e.*, an increase of pH to pH > 6.3) that causes the gelation of chitosan membranes. The flexible patternability of the photo-electrodes allows the generation of desired chitosan membrane patterns at a specific location, providing a great advantage over the electroaddressing of chitosan membranes using conventional metal microelectrodes. We demonstrate the ability to perform micropatterning of chitosan membranes with different shapes and sizes and with multiplexed micropatterning. Moreover, a multiplexed enzyme-based bioassay using chitosan membranes with entrapped enzymes was also successfully demonstrated through the electrodeposition of the chitosan membranes with various shapes/sizes and entrapping different enzymes. Each enzyme was easily identified by entrapment within different chitosan membrane shapes, creating a shape-coded multiplex assay system. As a model system, the simultaneous detection of glucose and ethanol was investigated by immobilizing GOX and AOX within chitosan membranes of different shapes. Because each chitosan membrane acted as an individual enzyme microreactor and allowed independent monitoring of different enzymatic reactions, the simultaneous detection of two different analytes was possible using the same fluorescence indicator. We anticipate that this simple, rapid, and flexible method for light-addressed electrodeposition of enzyme-entrapped chitosan membranes could be used to easily detect more than two analytes by depositing various chitosan membrane shapes with entrapped suitable receptor molecules.
